# Cross-cultural adaptation and validation of the brain injury vision symptom survey: bridging the gap with an Arabic version

**DOI:** 10.3389/fneur.2026.1759682

**Published:** 2026-01-20

**Authors:** Nawaf M. Almutairi, Amal Aloufi, Mohammed M. Alnawmasi, Abdullah Alawaji, Mohammad Alwosidi

**Affiliations:** Department of Optometry, College of Applied Medical Sciences, Qassim University, Buraydah, Saudi Arabia

**Keywords:** Arabic translation, BIVSS, cross-cultural adaptation, rehabilitation, symptom screening, traumatic brain injury, visual symptoms

## Abstract

**Background:**

Traumatic brain injury (TBI) often causes visual symptoms that hinder rehabilitation. The Brain Injury Vision Symptom Survey (BIVSS) is an established 28-item questionnaire for TBI-related visual symptoms, but it is only available in English. We aimed to create and validate an Arabic version to provide a culturally adapted tool for Arabic-speaking patients.

**Methods:**

The BIVSS was translated into Arabic using Beaton’s cross-cultural adaptation model (forward–backward translation, expert committee review, cognitive debriefing). Twenty-nine TBI patients completed the Arabic BIVSS twice, one month apart. Internal consistency was measured with Cronbach’s alpha. Test–retest reliability was assessed with an intraclass correlation coefficient [ICC(3,1)]. Agreement between test and retest was examined via Bland–Altman analysis (mean bias and 95% limits of agreement [LOA]). We also calculated the standard error of measurement (SEM) and the 95% minimal detectable change (MDC₉₅). Shapiro–Wilk tests were used to assess normality.

**Results:**

The Arabic BIVSS showed excellent internal consistency (Cronbach’s *α* = 0.897) and test–retest reliability (ICC(3,1) = 0.952, 95% CI [0.750, 0.984]). Bland–Altman analysis indicated a slight mean bias of −3.59 (test minus retest), with 95% LOA from −11.71 to 4.54. No proportional bias was observed. The SEM was 2.93, and MDC₉₅ was 8.12 points. Score distributions were approximately normal (Shapiro–Wilk *p* = 0.024 at baseline, 0.083 at retest).

**Conclusion:**

The Arabic BIVSS is a reliable and valid instrument for assessing TBI-related visual symptoms. This cross-culturally adapted version can facilitate clinical screening and research in Arabic-speaking populations. However, given the small sample size and lack of a control group, further research is required to establish population-specific cut-off values and assess construct validity and responsiveness.

## Introduction

Traumatic Brain Injury (TBI) represents a pressing and persistent global public health challenge, inflicting a significant burden of mortality and long-term disability. The Global Burden of Disease 2021 study estimates that TBI accounted for 20.83 million incident cases and 37.92 million prevalent cases worldwide in 2021 (1, 2).

Among the most common yet frequently overlooked sequelae of TBI is a “hidden epidemic” of visual dysfunction (3). The visual system’s extensive neural integration renders it exceptionally vulnerable to the diffuse axonal and neurometabolic injuries associated with brain trauma. Consequently, an estimated 69 to 90% of TBI patients report debilitating visual symptoms (4, 5). This clinical profile includes blurred or double vision (diplopia), convergence insufficiency, accommodative (focusing) dysfunction, oculomotor impairments, and photophobia (4, 6–9). These visual deficits are not benign; they profoundly impair reading, mobility, and daily function, and can significantly impede overall TBI rehabilitation efforts (10–12).

To identify and quantify these subjective, patient-experienced impairments, clinicians and researchers primarily rely on the Brain Injury Vision Symptom Survey (BIVSS), a 28-item questionnaire developed by Laukkanen et al. (13). The BIVSS is recognized in the field as the only validated, vision-specific questionnaire designed explicitly for TBI patients and serves as a critical screening tool in clinical practice. However, this essential instrument was developed in English, rendering it inaccessible to the millions of TBI patients in the Arabic-speaking world. This creates a significant gap in clinical practice, as the simple, direct translation of the BIVSS is insufficient and yields psychometrically invalid results without a formal cross-cultural adaptation process.

The clinical need for a validated Arabic version of BIVSS is underscored by the region’s specific epidemiological context. Recent (2025) analysis of GBD 2021 data identifies Saudi Arabia as a notable outlier, with one of the highest TBI incidence rates globally at 681 cases per 100,000 people (1). This figure is substantially higher than previously cited estimates (14). Furthermore, this high incidence is compounded by a worsening regional trend; recent 2025 analyses confirm that TBI-related disability rates in the area are increasing, in direct contrast to global declines (1). This significant public health burden is met by a documented “knowledge gap” within the local clinical community. A 2025 survey of optometrists in Saudi Arabia revealed that only 16.8% possess high competency in managing TBI-related visual disorders (15). Consequently, a fundamental disparity exists: a high-risk population faces a clinical environment that lacks the validated screening instruments required for the effective detection and management of TBI-related visual dysfunction.

To address this critical, multifaceted gap in clinical practice, epidemiology, and regional public health, the present study was designed. We aimed to formally translate, culturally adapt, and psychometrically validate the Brain Injury Vision Symptom Survey (BIVSS) for use in Arabic-speaking populations. This work provides the first validated, TBI-specific visual symptom screening tool for clinicians and researchers in the Arabic-speaking world, establishing a foundation for improved detection, rehabilitation, and patient-centered care.

## Methods

### Study design and setting

This study employed a prospective, cross-sectional validation design. The protocol was approved by the Committee of Research Ethics at Qassim University and adhered to the principles of the Declaration of Helsinki. All participants provided written informed consent prior to inclusion. Data were collected from individuals at clinical sites affiliated with hospitals in the Qassim region, Saudi Arabia.

### Participants

Participants were recruited through convenience sampling from clinical sites affiliated with hospitals in the Qassim region, Saudi Arabia. The sample consisted of 31 Arabic-speaking adults (aged ≥18 years) with a clinically confirmed history of mild to severe traumatic brain injury (TBI) based on Glasgow Coma Scale scores, as determined by neurological or medical records. Two participants did not complete any BIVSS items and were therefore excluded from the psychometric analyses, resulting in a final analytic sample of 29 TBI participants. Exclusion criteria included significant ocular pathology unrelated to TBI, severe cognitive impairment, psychiatric illness, or inability to complete the questionnaire independently. Demographic information, such as age, gender, and time since injury, was recorded.

Sample size was planned for test–retest reliability using an ICC hypothesis-testing approach (*ρ*₀ = 0.70, ρ₁ = 0.90, power = 80%, k = 2). Using the Mondal et al. (16) sample-size application and adopting the most conservative estimate across methods (*n* = 24; Z_Ze_), our final analytic sample (*n* = 29) exceeded the required size. A non-TBI Arabic-speaking control group was not included in this initial reliability phase; future studies should include matched controls to establish normative values and population-specific cut-off scores.

### Translation and cross-cultural adaptation

The translation process followed internationally recognized guidelines proposed by Guillemin et al. (17) and Beaton et al. (18) for adapting patient-reported outcome measures and involved following structured stages:

#### Stage I: forward translation

Two independent forward translations of the original English BIVSS into Arabic were produced by bilingual optometrists who were native Arabic speakers and were familiar with TBI-related visual disorders. Each translator worked independently and prepared a written report documenting translation choices and any expressions that proved difficult to render into Arabic.

#### Stage II: synthesis of translations

In a consensus meeting, the two forward translations (T1 and T2) were compared item by item. Discrepancies were discussed by an expert committee comprising two optometrists, one vision scientist, and one professional linguist, and a single reconciled version (T-12) was produced.

#### Stage III: back-translation

The synthesized Arabic version (T-12) was then independently back-translated into English by two professional translators who were English speakers and who were blinded to the original BIVSS and had no clinical background. These back-translations (BT1 and BT2) served as a validity check to identify ambiguous wording or conceptual drift in the forward translation.

#### Stage IV: expert committee review

A multidisciplinary expert committee (two optometrists, one vision scientist, one linguist, and two back-translators) reviewed the original BIVSS, the forward translations (T1, T2), the synthesized version (T-12), and the two back-translations (BT1, BT2). The committee evaluated equivalence at four levels: semantic (word meaning and grammar), idiomatic (colloquial expressions), experiential (the relevance of examples to daily life in Arabic-speaking contexts), and conceptual (the underlying construct). Where needed, wording was refined to ensure that items, instructions, and response options were clear and culturally appropriate while maintaining the intent of the original instrument. This process yielded a pre-final Arabic version of the BIVSS.

#### Stage V: pre-testing (cognitive debriefing)

The pre-final BIVSS-Arabic was pilot-tested with 10 Arabic-speaking adults with TBI. Participants completed the questionnaire and were then interviewed about their understanding of each item, the clarity of wording, and the relevance of examples. Feedback was used to identify any residual comprehension or cultural-fit issues. Minor linguistic refinements (e.g., simplifying phrasing and replacing rare terms with commonly used equivalents) were made without altering the underlying concepts, resulting in the final Arabic version of the BIVSS-Arabic.

Permission to translate and adapt the BIVSS was obtained from the original developers before initiating the process, and all documentation from each stage of adaptation was retained to allow independent appraisal of the translation procedure.

### Questionnaire structure and scoring

The original BIVSS consists of 28 items categorized into eight subdomains: visual clarity, visual comfort, doubling, light sensitivity, dry eyes, depth perception, peripheral vision, and reading. Each item is scored on a 5-point Likert scale from 0 (“never”) to 4 (“always”), where higher scores indicate more frequent or severe visual symptoms. The total BIVSS score was calculated as the sum of all item scores, and subscale scores were computed by averaging the items within each domain.

### Data collection

All participants completed the finalized Arabic BIVSS under standardized conditions. Investigators provided instructions and ensured a complete understanding of each question. To assess test–retest reliability, all participants from the TBI group completed the questionnaire again 1 month after the initial administration. The interval was chosen to minimize memory effects while preventing clinical changes in visual symptoms.

### Statistical analysis

Data were screened for completeness; participants with no item responses at either time point were excluded. For each time point, a total BIVSS score was computed by summing the 28 items. Descriptive statistics (mean, SD, minimum, maximum) were obtained for test and retest totals. Distributional shape was assessed with the Shapiro–Wilk test. Floor/ceiling effects were evaluated as the percentage at the theoretical minimum (0) and maximum (112); values >15% were considered problematic. Internal consistency of the total score at baseline was assessed with Cronbach’s alpha (*α*), accompanied by corrected item–total correlations and “alpha if item deleted.” Test–retest reliability of the total score was evaluated with the intraclass correlation coefficient, with 95% confidence intervals, employing a two-way mixed-effects model with absolute agreement and single-measurement definition [ICC(3,1)]. This model is appropriate for test–retest reliability because the measurement occasions are fixed (the same instrument administered twice under the same protocol) rather than randomly sampled. Absolute agreement was selected because test–retest reliability requires that repeated administrations yield interchangeable scores, not merely correlated scores (19). Agreement was quantified using a Bland–Altman analysis: the mean difference (bias), the SD of the differences, and 95% limits of agreement (LOA = bias ± 1.96 × SD) were reported. Proportional bias was examined by regressing the difference on the mean. Measurement-error indices were derived as SEM = SD(diff)/√2 and MDC95 = 1.96 × √2 × SEM (smallest individual change beyond error). Statistical analyses were conducted using IBM SPSS Statistics, version 31.0.1.0(49) (IBM Corp., Armonk, NY, United States).

## Results

### Subject profile

A total of 31 Arabic-speaking adults with TBI were enrolled in the study. Two participants did not complete any BIVSS items and were excluded from psychometric analyses, yielding a final analytic sample of 29 participants with complete baseline and retest BIVSS data. The mean age of this analytic sample was 28.14 years (SD = 11.61; range = 18–60 years), and 62.1% (*n* = 18) were female. Time since injury ranged from 1 day to 26 years, with a mean of approximately 6.8 years (mean = 2,494 days, SD = 2,745 days). Where severity information was available, cases included mild, moderate, and more severe injuries, as well as emergency presentations, reflecting the heterogeneous nature of TBI in clinical practice.

### Descriptive statistics and distribution

Total BIVSS scores at baseline ranged from 2 to 75 with M = 28.66, SD = 17.17 (*n* = 29). At retest, totals ranged 6–80 with M = 32.24, SD = 17.53 (*n* = 29). Because observed minima and maxima were well within the theoretical range (0–112), no floor or ceiling effects were evident. The Shapiro–Wilk test indicated a slight departure from normality at baseline (W = 0.916, *p* = 0.024) and no significant deviation at retest (W = 0.937, *p* = 0.083); given *n* = 29 and the robustness of reliability/agreement metrics, parametric procedures were retained (Table 1).

**Table 1:** Participant characteristics.

Characteristic	Value
*N*	29
Age, years	28.14 ± 11.61 (range 18–60)
Gender, *n* (%)	Female 18 (62.1%); Male 11 (37.9%)
Time since injury, years	6.83 ± 7.51 (range ~0–26)

### Internal consistency (baseline)

The Arabic BIVSS total score showed excellent internal consistency (Cronbach’s *α* = 0.897, 28 items). Corrected item–total correlations spanned 0.145–0.691; two items were at or below 0.30 (Item 16 = 0.145; Item 4 = 0.295). Removing any single item changed α by <0.01 (maximum α if deleted = 0.902 for Item 16), so all items were retained (Table 2).

**Table 2:** Reliability and agreement of the Arabic BIVSS (total score).

Metric	Estimate	95% CI	Interpretation
Internal consistency (Cronbach’s *α*)	0.897	–	Excellent internal consistency
ICC(3,1) (two-way mixed, absolute agreement, single measures)	0.952	[0.750, 0.984]	Excellent test–retest reliability
ICC(3,2) (average measures)	0.975	[0.857, 0.992]	Excellent reliability (average measures)
Bias (test − retest)	−3.59	–	Retest slightly higher than test
SD of differences	4.14	–	Variability of test–retest differences
95% limits of agreement	−11.71 to 4.54	—	Bland–Altman LOA
SEM	2.93	—	Measurement error
MDC95	8.12	—	Smallest real change

α = Cronbach’s alpha; ICC = intraclass correlation coefficient; SEM = SD(diff)/√2; MDC95 = 1.96·√2·SEM.

### Test–retest reliability

Test–retest reliability for the total score was excellent: ICC(3,1) = 0.952, 95% CI [0.750, 0.984], *F*(28, 28) = 69.10, *p* < 0.001. For reference, the average-ICC was 0.975 (95% CI [0.857, 0.992]) (Table 2).

### Agreement and measurement error

**Figure 1:**
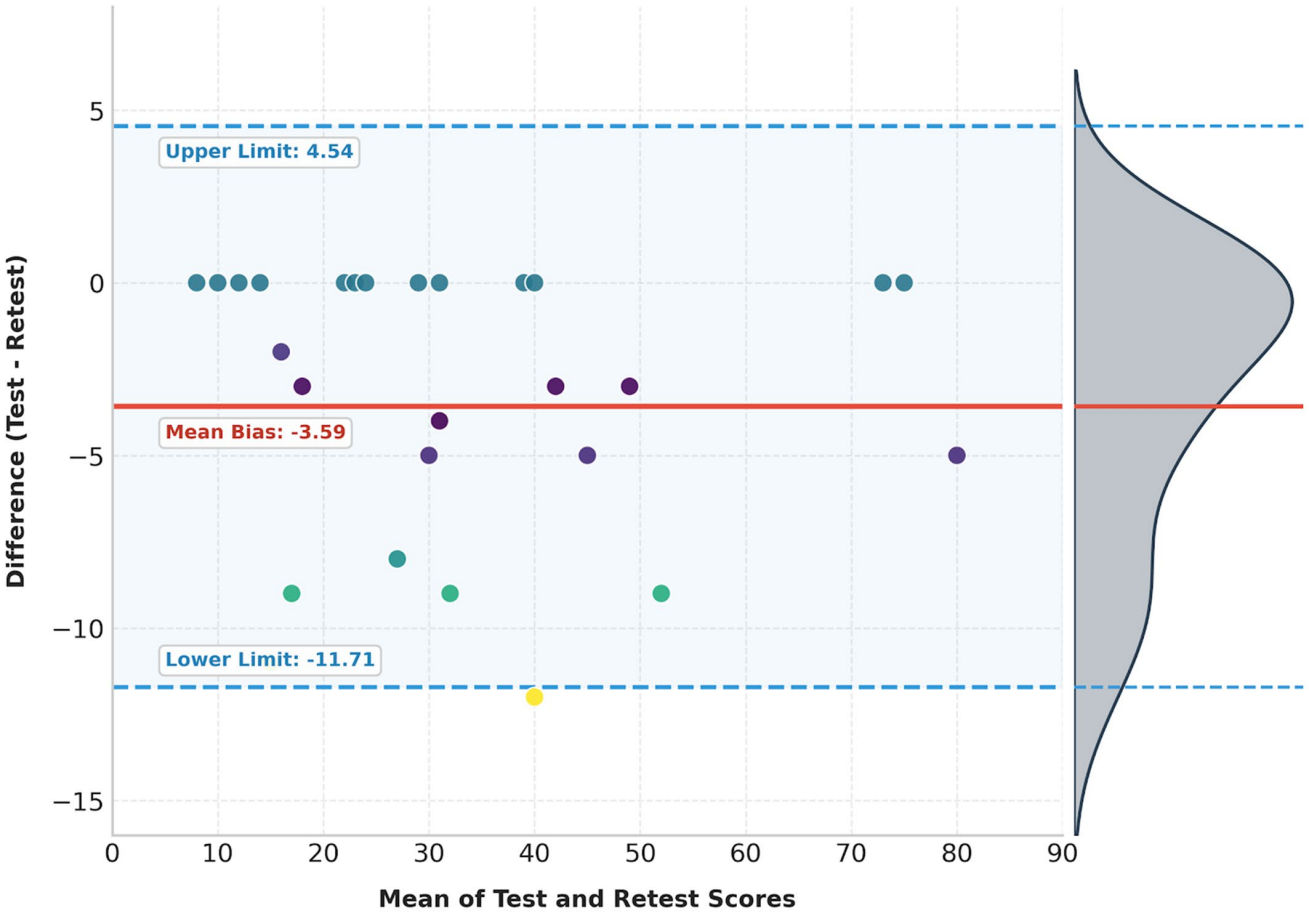
Agreement analysis between test and retest measurements. The scatter plot displays the difference between scores plotted against their mean. Data points are color-coded based on their deviation from the mean bias (solid red line, −3.59). The shaded region bounded by dashed blue lines represents the 95% limits of agreement (−11.71 to 4.54). The marginal density curve on the right illustrates the distribution of differences, highlighting the clustering of data points relative to the bias.

Bland–Altman analysis showed a small bias of −3.59 points (test − retest), with SD (diff) = 4.14; the 95% LOA were −11.71 to 4.54. Regression of the difference on the mean indicated no proportional bias (slope = −0.021, *p* = 0.647). From SD(diff), SEM was 2.93 points, and MDC95 was ≈ 8.12 points, indicating that individual changes > 8 points on the total score are likely to reflect actual change beyond measurement error (Figure 1; Table 2).

## Discussion

This validation of the Arabic BIVSS indicates that the survey retains excellent psychometric properties in translation. The total scale showed high internal consistency (Cronbach’s *α* = 0.897), comparable to the original English BIVSS development, which reported a similar reliability level (Rasch person reliability ~0.90) and effective discrimination of TBI-related visual symptoms (13). In our sample of 29 TBI patients, test–retest reliability was likewise excellent (ICC(3,1) = 0.952, 95% CI [0.750–0.984]), in line with prior findings for the English version (e.g., *r* ≈ 0.91 over two administrations) (20). These reliability metrics mirror those of other vision-specific patient-reported outcome measures adapted to Arabic. For instance, the Arabic version of the NEI VFQ-25 visual function questionnaire demonstrated Cronbach’s *α* up to 0.91 with test–retest ICC ~ 0.79 (21). The Arabic BIVSS thus performs on par or better in terms of consistency and reproducibility, bolstering confidence that the instrument’s items are understood similarly by Arabic-speaking TBI patients.

Establishing an Arabic-language vision symptom survey has important implications for TBI care in Arabic-speaking populations. The consistent pattern of elevated symptom scores in the TBI aligns with existing literature that documents the high prevalence of visual disturbances following brain trauma (9, 22). These symptoms, including diplopia, visual discomfort, reading difficulties, and accommodative issues, are often underrecognized despite their significant impact on quality of life (23, 24).

The findings reinforce the neurological basis of visual dysfunction in TBI, stemming from the vulnerability of the oculomotor and visual processing systems to diffuse axonal injury, especially in mild-to-moderate cases (6, 25). The broad range of symptom severity reported by participants suggests substantial heterogeneity in visual outcomes, which further justifies the use of symptom-specific screening instruments such as the BIVSS.

The availability of the BIVSS in Arabic can improve systematic screening for such issues. Routine use of this survey in neurology or rehabilitation clinics could help non-specialist providers identify patients with significant vision-related symptoms who might otherwise be overlooked. In the context of our sample, even mild TBI patients were able to self-report a range of symptoms. Having a quantifiable Arabic symptom score enables clearer communication and referral: patients reporting higher total BIVSS scores and/or symptom patterns that interfere with reading, mobility, or daily activities may warrant referral to optometrists or neuro-ophthalmologists for comprehensive visual evaluation. However, because Arabic-specific normative data and diagnostic cut-off values were not established in the present study (no healthy control group), “higher” scores should be interpreted as greater symptom burden rather than a validated threshold indicating that a patient is definitively “symptomatic” or requires intervention. This fills a notable gap in multidisciplinary TBI care, as previously highlighted by the developers of BIVSS. Moreover, an Arabic BIVSS can facilitate research in Arab countries by providing a standardized outcome measure for clinical trials or epidemiological studies on TBI-related visual dysfunction, analogous to how the English BIVSS has been used to characterize vision symptom profiles in TBI.

Several strengths of the present study support the robustness of our findings. We followed established guidelines for cross-cultural adaptation, including translation and back-translation, expert review, and pilot testing, to ensure the Arabic BIVSS is conceptually equivalent to the original (18). The resulting instrument demonstrated strong psychometric performance despite the modest sample size, suggesting that the item content was clear and relevant to our participants. However, we acknowledge certain limitations. First, our study lacked a non-TBI Arabic-speaking control group, which precluded evaluation of discriminative validity and prevented the derivation of Arabic normative values and a population-specific clinical cut-off score. The original BIVSS validation included a control group of 157 subjects and proposed a cut-off score of 31 for identifying symptomatic patients; in the absence of an Arabic control cohort, we cannot assume that this threshold generalizes to Arabic-speaking populations, and clinicians should interpret total scores as an index of symptom burden rather than a definitive diagnostic classification. Future studies should prioritize recruitment of a healthy Arabic-speaking control cohort and apply criterion/diagnostic validity methods (e.g., ROC-based thresholds) to establish clinically meaningful cut-offs and referral guidance. Second, the BIVSS is intentionally vision-focused and does not assess broader quality-of-life domains (e.g., activity limitation, mobility, general symptoms, convenience, health concerns, emotional, social, and economic impacts); therefore, it should be considered a targeted screening tool for vision-related symptom burden and ideally complemented by broader outcome measures when comprehensive quality-of-life assessment is required. Third, the sample size was relatively small and drawn from a single TBI population, limiting our ability to perform advanced analyses such as factor analysis or Rasch modeling to confirm dimensional structure post-translation. Fourth, we focused on reliability and basic content validity but did not formally assess construct validity. Future research should examine how Arabic BIVSS scores correlate with external measures—for example, general post-concussion symptom scales or objective clinical findings (e.g., vestibulo-ocular test results)—to establish convergent validity. Additionally, longitudinal studies could determine the instrument’s responsiveness to interventions (e.g., vision rehabilitation therapy) in Arabic-speaking TBI patients.

## Conclusion

In summary, the Arabic version of the Brain Injury Vision Symptom Survey exhibits high internal consistency and excellent test–retest reliability, on par with or exceeding that of established vision-related surveys. The availability of this validated Arabic instrument is an important advancement, as it allows clinicians and researchers to capture the visual symptoms of Arabic-speaking TBI patients accurately. This tool can facilitate better screening, tracking, and ultimately management of post-traumatic visual problems in a large patient population. Further research is warranted to explore the instrument’s construct validity and responsiveness to change, which will augment its utility in both clinical and research settings.

## Data Availability

The raw data supporting the conclusions of this article will be made available by the authors, without undue reservation.
