# Supplementary material for: Cross-cultural adaptation and validation of the brain injury vision symptom survey: bridging the gap with an Arabic version
Source: Front Neurol. 2026 Jan 20;17:1759682. doi: 10.3389/fneur.2026.1759682 (PMC12864060; doi:10.3389/fneur.2026.1759682)
Supplement: Supplementary file 1 [file Table_1.docx]

**Appendix. A**

The original English version of brain injury vision symptoms score (BIVSS).
