# Supplementary material for: Cross-cultural adaptation and validation of the brain injury vision symptom survey: bridging the gap with an Arabic version
Source: Front Neurol. 2026 Jan 20;17:1759682. doi: 10.3389/fneur.2026.1759682 (PMC12864060; doi:10.3389/fneur.2026.1759682)
Supplement: Supplementary file 2 [file Table_2.docx]

**Appendix. B**

The Translated Arabic version of brain injury vision symptoms survey (BIVSS-Arabic).

**استبيان مسح أعراض الرؤية لإصابات الدماغ**

اسم المشارك: ......................................................... تاريخ اليوم: ...........................

إصابة الدماغ كانت قبل ....................يوم/شهر/سنة. العمر: ................سنة.

☐ لدي تشخيص طبي لإصابة الدماغ (حدد المربع إذا كان صحيحاً). سبب الإصابة: _______________

☐ تعرضت لإصابة دماغ ولكن لم يتم تشخيصي طبياً (حدد المربع إذا كان صحيحاً) _______________

☐ أبداً لم أتعرض للإصابة الدماغ.

يرجى وضع دائرة حول المربع الأنسب الذي يطابق ملاحظاتك.

| **قائمة التحقق من الأعراض**  كم مرة يحدث كل عرض مدرج أدناه؟  يرجى وضع دائرة حول رقم على اليمين. | | **أبدا** | **نادرا** | **احيانا** | **بشكل متكرر** | **دوما** |
| --- | --- | --- | --- | --- | --- | --- |
| **وضوح الرؤية** | | | | | | |
| 1 | الرؤية البعيدة ضبابية وغير واضحة - حتى مع العدسات | 0 | 1 | 2 | 3 | 4 |
| 2 | الرؤية القريبة ضبابية وغير واضحة - حتى مع العدسات | 0 | 1 | 2 | 3 | 4 |
| 3 | وضوح الرؤية يتغير أو يتقلب خلال النهار | 0 | 1 | 2 | 3 | 4 |
| 4 | الرؤية الليلية ضعيفة / لا أستطيع الرؤية جيدا اثناء القيادة ليلا | 0 | 1 | 2 | 3 | 4 |
| **الراحة البصرية** | | | | | | |
| 5 | عدم الراحة في العين / الم في العينين / إجهاد العين | 0 | 1 | 2 | 3 | 4 |
| 6 | صداع أو دوخة بعد استخدام العين | 0 | 1 | 2 | 3 | 4 |
| 7 | إرهاق في العين / متعب جدا بعد استخدام العينين طوال اليوم | 0 | 1 | 2 | 3 | 4 |
| 8 | اشعر "بالضغط" حول العينين | 0 | 1 | 2 | 3 | 4 |
| **الرؤية المزدوجة** | | | | | | |
| 9 | رؤية الأشياء مزدوجة - خاصة عند التعب | 0 | 1 | 2 | 3 | 4 |
| 10 | تلجأ الي اغلاق أو تغطية احدي العينين لترى بوضوح. | 0 | 1 | 2 | 3 | 4 |
| 11 | تصبح الكتابة مرة واضحة ومرة غير واضحة أثناء القراءة. | 0 | 1 | 2 | 3 | 4 |
| **التحسس من الضوء** | | | | | | |
| 12 | الإضاءة الداخلية العادية غير مريحة – تسبب الكثير من الوهج. | 0 | 1 | 2 | 3 | 4 |
| 13 | الضوء الخارجي ساطع جدا – تحتاج أن تستخدم النظارات الشمسية. | 0 | 1 | 2 | 3 | 4 |
| 14 | اضاءة مصابيح الفلوريسنت الداخلية مزعجة. | 0 | 1 | 2 | 3 | 4 |
| **جفاف العين** | | | | | | |
| 15 | الشعور بالجفاف في العينين واللسع | 0 | 1 | 2 | 3 | 4 |
| 16 | تحدق في الفضاء دون ان ترمش. | 0 | 1 | 2 | 3 | 4 |
| 17 | تلجأ أن تفرك العينين كثيرا | 0 | 1 | 2 | 3 | 4 |
| **إدراك العمق** | | | | | | |
| 18 | سوء تقدير مكان وجود الأشياء في الواقع | 0 | 1 | 2 | 3 | 4 |
| 19 | انعدام الثقة أثناء المشي / فقدان الخطوات / التعثر | 0 | 1 | 2 | 3 | 4 |
| 20 | سوء الكتابة اليدوية (التباعد/ الحجم / الوضوح) | 0 | 1 | 2 | 3 | 4 |
| **الرؤية المحيطية** | | | | | | |
| 21 | الرؤية الجانبية مشوشة / الأجسام تتحرك أو تغير موضعها | 0 | 1 | 2 | 3 | 4 |
| 22 | الشيئ الذي تراه في الامام مباشر، غالبا لايكون في الامام مباشره | 0 | 1 | 2 | 3 | 4 |
| 23 | تتجنب الازدحام/ لا تستطيع تحمل الأماكن المزدحمة بصريا. | 0 | 1 | 2 | 3 | 4 |
| **القراءة** | | | | | | |
| 24 | فترة انتباه قصيرة / يتشتت انتباهك بسهولة عند القراءة | 0 | 1 | 2 | 3 | 4 |
| 25 | صعوبة / بطء في القراءة والكتابة | 0 | 1 | 2 | 3 | 4 |
| 26 | ضعف فهم القراءة والاستيعاب / لا أتذكر ما تمت قراءته | 0 | 1 | 2 | 3 | 4 |
| 27 | اختلاط الكلمات / تخطي الكلمات أثناء القراءة | 0 | 1 | 2 | 3 | 4 |
| 28 | تفقد المكان / تضطر إلى استخدام الأصابع للمتابعة عند القراءة | 0 | 1 | 2 | 3 | 4 |
