# Supplementary material for: Cross-cultural adaptation and validation of the brain injury vision symptom survey: bridging the gap with an Arabic version
Source: Front Neurol. 2026 Jan 20;17:1759682. doi: 10.3389/fneur.2026.1759682 (PMC12864060; doi:10.3389/fneur.2026.1759682)
Supplement: Supplementary file 3 [file Table_3.docx]

**Appendix. C**

This table presents the item-level statistics for the Arabic BIVSS total score at baseline, derived from the reliability analysis of the 28 items.

| Item # | Item Description (Domain) | Corrected Item-Total Correlation | Cronbach’s Alpha if Item Deleted |
| --- | --- | --- | --- |
| 1 | Distance vision blurred and not clear – even with the lenses (Eyesight Clarity) | 0.339 | 0.897 |
| 2 | Near vision blurred and not clear – even with lenses (Eyesight Clarity) | 0.360 | 0.896 |
| 3 | Clarity of vision changes of fluctuates during the day (Eyesight Clarity) | 0.445 | 0.894 |
| 4 | Poor night vision / can’t see well to drive at night (Eyesight Clarity) | 0.295 | 0.898 |
| 5 | Eye discomfort / sore eyes / eyestrain (Visual Comfort) | 0.637 | 0.890 |
| 6 | Headaches or dizziness after using eyes (Visual Comfort) | 0.331 | 0.897 |
| 7 | Eye fatigue / very tired after using eyes all day (Visual Comfort) | 0.553 | 0.892 |
| 8 | Feel “pulling” around the eyes (Visual Comfort) | 0.593 | 0.891 |
| 9 | Double vision – especially when tired (Doubling) | 0.399 | 0.895 |
| 10 | Have to close or cover one eye to see correctly (Doubling) | 0.607 | 0.891 |
| 11 | Print moves in and out of focus when reading (Doubling) | 0.326 | 0.897 |
| 12 | Normal indoor lighting is uncomfortable – too much glare (Light Sensitivity) | 0.444 | 0.894 |
| 13 | Outdoor light too bright – have to use sunglasses (Light Sensitivity) | 0.484 | 0.894 |
| 14 | Indoors fluorescent lighting is bothersome or annoying (Light Sensitivity) | 0.596 | 0.891 |
| 15 | Eyes feel “dry” and sting (Dry Eyes) | 0.609 | 0.891 |
| 16 | “Stare” into space without blinking (Dry Eyes) | 0.145 | 0.902 |
| 17 | Have to rub eyes a lot (Dry Eyes) | 0.301 | 0.898 |
| 18 | Clumsiness / misjudge where objects really are (Depth Perception) | 0.540 | 0.892 |
| 19 | Lack of confidence walking / missing steps / stumbling (Depth Perception) | 0.476 | 0.894 |
| 20 | Poor handwriting (spacing, size, legibility) (Depth Perception) | 0.583 | 0.892 |
| 21 | Side vision distorted / objects move or change position (Peripheral Vision) | 0.621 | 0.891 |
| 22 | What looks straight ahead – isn’t always straight ahead (Peripheral Vision) | 0.524 | 0.894 |
| 23 | Avoid crowds / can’t tolerate “visually-busy” places (Peripheral Vision) | 0.461 | 0.894 |
| 24 | Short attention span / easily distracted when reading (Reading) | 0.447 | 0.894 |
| 25 | Difficulty / slowness with reading and writing (Reading) | 0.471 | 0.894 |
| 26 | Poor reading comprehension / can’t remember what was read (Reading) | 0.547 | 0.892 |
| 27 | Confusion of words / skip words during reading (Reading) | 0.691 | 0.891 |
| 28 | Lose place / have to use finger to not lose place when reading (Reading) | 0.461 | 0.894 |
